# Trans-abdominal *in vivo* placental vessel occlusion using High Intensity Focused Ultrasound

**DOI:** 10.1038/s41598-018-31914-4

**Published:** 2018-09-11

**Authors:** Caroline J. Shaw, Ian Rivens, John Civale, Kimberley J. Botting, Gail ter Haar, Dino A. Giussani, Christoph C. Lees

**Affiliations:** 1 Department of Physiology, Development and Neuroscience, University of Cambridge, Cambridge, CB2 3EG UK; 2 Institute of Reproductive and Developmental Biology, Imperial College London, London, W12 0HS UK; 3 Joint Department of Physics, Institute of Cancer Research, Sutton, London SM2 5NG UK; 4 Cardiovascular Strategic Research Initiative, University of Cambridge, Cambridge, UK; 5 Department of Obstetrics and Gynaecology, University Hospitals Leuven, 3000 Leuven, Belgium

## Abstract

Pre-clinically, High Intensity Focused Ultrasound (HIFU) has been shown to safely and effectively occlude placental blood vessels in the acute setting, when applied through the uterus. However, further development of the technique to overcome the technical challenges of targeting and occluding blood vessels through intact skin remains essential to translation into human studies. So too does the assessment of fetal wellbeing following this procedure, and demonstration of the persistence of vascular occlusion. At 115 ± 10 d gestational age (term~147 days) 12 pregnant sheep were exposed to HIFU (n = 6), or to a sham (n = 6) therapy through intact abdominal skin (1.66 MHz, 5 s duration, *in situ* I_SPTA_ 1.3–4.4 kW.cm^−2^). Treatment success was defined as undetectable colour Doppler signal in the target placental vessel following HIFU exposures. Pregnancies were monitored for 21 days using diagnostic ultrasound from one day before HIFU exposure until term, when post-mortem examination was performed. Placental vessels were examined histologically for evidence of persistent vascular occlusion. HIFU occluded 31/34 (91%) of placental vessels targeted, with persistent vascular occlusion evident on histological examination 20 days after treatment. The mean diameter of occluded vessels was 1.4 mm (range 0.3–3.3 mm). All pregnancies survived until post mortem without evidence of significant maternal or fetal iatrogenic harm, preterm labour, maternal or fetal haemorrhage or infection. Three of six ewes exposed to HIFU experienced abdominal skin burns, which healed without intervention within 21 days. Mean fetal weight, fetal growth velocity and other measures of fetal biometry were not affected by exposure to HIFU. Fetal Doppler studies indicated a transient increase in the umbilical artery pulsatility index (PI) and a decrease in middle cerebral artery PI as a result of general anaesthesia, which was not different between sham and treatment groups. We report the first successful application of fully non-invasive HIFU for occlusion of placental blood flow in a pregnant sheep model, with a low risk of significant complications. This proof of concept study demonstrates the potential of this technique for clinical translation.

## Introduction

High Intensity Focused Ultrasound (HIFU) is an established non-invasive therapeutic technique with applications in particularly in oncology and neurosurgery. In gynaecology, the best recognised use of HIFU is for the ablation of uterine fibroids, under MRI guidance. HIFU has a potential application as a method of placental and fetal vascular occlusion, or in fetal soft tissue ablation^1–7^.

The use of HIFU to occlude blood vessels has been suggested as non-invasive treatment for fetal conditions where access to blood vessels is restricted, and conventional techniques such as open or minimal access surgery are required, hence the risks of intervening invasively are high. These applications include twin-twin transfusion syndrome (TTTS), twin reversed arterial perfusion (TRAP) sequence, bronchopulmonary sequestration and sacrococcygeal teratoma^8,9^. While fetal and placental soft tissue ablation using ultrasound guided HIFU has been demonstrated in human patients^1,2^ and animal models^4,5,10^, selective placental and fetal vascular occlusion using HIFU is less well developed. Our group has previously reported the use of ultrasound guided HIFU to selectively and consistently occlude placental vessels in a pregnant sheep model using a transuterine approach via laparotomy. This demonstrated short term (up to 4 hours) placental vascular occlusion and fetal survival following the application of HIFU; cardiovascular and metabolic invasive assessment of fetal responses suggested that the technique was safe in the acute setting^3^. There was one instance of placental vessel haemorrhage.

These findings, though promising, did not fully address some specific challenges related to HIFU mediated vascular occlusion. Firstly, before translation into human studies can be justified, evidence as to fetal survival and wellbeing beyond 4 hours following HIFU placental vascular occlusion is desirable and was not described. Secondly, the persistence of the vascular occlusion achieved using our techniques (occlusive clot) has not been demonstrated, as vascular lumen were not obliterated. Finally, occlusion of vessels with HIFU typically requires higher energy levels than soft tissue ablation^8^. However, if an inappropriate acoustic window is used, ultrasonic energy may be scattered or reflected, preventing transmission and causing heating in unpredictable locations, and causing burns. The skin is particularly vulnerable to this as it has a higher absorption coefficient compared to other soft tissues, and thermal energy has a tendency to accumulate in subcutaneous fat^11^. Hence, it remains important to demonstrate that a HIFU treatment protocol can safely deliver sufficient energy into the intrauterine space to achieve successful vascular occlusion with transducers placed external to the skin, not just the uterus, to justify its description as a non-invasive technique.

Therefore, in this next-stage study, the objective was to further develop the techniques used previously. Specifically, the aim was to test the efficacy of using ultrasound-guided HIFU applied through intact maternal abdominal skin to selectively occlude placental blood vessels in pregnant sheep, and to investigate whether persistent vascular occlusion could be achieved. In so doing, we describe maternal and fetal recovery from HIFU placental vascular occlusion and describe side-effects.

## Methods

All procedures were performed in accordance with the UK Animals (Scientific Procedures) Act 1986 and were approved by the Ethical Review Committee of the University of Cambridge.

Twelve singleton Welsh Mountain sheep fetuses at 115 ± 10 d gestational age (median ± range, term ~147 days) were used. Six ewes were exposed to HIFU (animals 1–6 chronologically) and six were sham exposed (animals 7–12). Maternal and fetal wellbeing was monitored before, during and after HIFU or sham exposures, over a total of 21 days. A timeline of experimental procedures and follow-up is shown in Figure s1.

### Assessment of baseline of materno-fetal wellbeing prior to HIFU/sham exposures

Prior to HIFU or sham exposures of placental vasculature, materno-fetal ultrasound was performed in un-anaesthetised animals to establish a baseline for fetal biometry and materno-fetal Doppler studies (“day −1”). The methods used for Doppler evaluation of maternal and fetal vessels were based on good practice guidelines published for use in human pregnancy^12^. Fetal biometry was also measured during this assessment, based on methods described in the published literature.

The umbilical cord was identified using colour flow Doppler, and the arterial pulsatility index (PI) was measured with pulsed-wave Doppler, using auto-tracing of the best three consecutive waveforms. Umbilical artery (UA) Doppler was performed in all cases in a free loop of umbilical cord near the cord insertion. This was done to ensure that the sampling was performed in the full umbilical cord downstream of where all allantoic vessels have joined.

Middle cerebral artery (MCA) Doppler was performed in an axial section of the fetal head at the level of the sphenoid wings, with colour flow mapping used to identify the circle of Willis. Sampling was undertaken using pulsed-wave Doppler within the proximal third of the vessel; no angle correction was applied and the angle of insonation was kept as close to 0° as possible and in all cases less than 20^0^.

Ductus venosus (DV) Doppler was performed in either an oblique transverse section through the upper abdomen or in the mid-sagittal longitudinal section, depending on fetal positioning. Using B-mode imaging, the vessel was first identified between the umbilical vein and the inferior vena cava and was confirmed as having characteristic aliasing on colour flow Doppler, and then sampled with pulsed-wave Doppler.

The maternal uterine artery was identified with colour flow Doppler where it crossed the external iliac artery, and the pulsed-wave Doppler gate was placed within 1 cm of this crossover point. Measurements were taken bilaterally, and PI was measured by auto-tracing of the best three consecutive waveforms; values quoted are an average of both sides.

Fetal biparietal diameter (BPD) was estimated as the widest linear distance between parietal bones behind the proximal end of the supra-orbital processes with the fetal head in axial view^13^. The abdominal circumference (AC) was measured in a transverse circular view of the fetal abdomen at the level of the 12^th^ thoracic rib, just above the insertion of the umbilical cord, using an ellipse^14^. The femur length (FL) was measured as the calcified length of the bone with the angle of insonation perpendicular to the shaft of the bone^13^. Fetal weight was estimated using the Hadlock (BPD-AC-FL) formula optimised for use in human pregnancy^15^.

### Induction and maintenance of general anaesthesia

Animals were fasted for 24 hours before the induction of anaesthesia, which was required to allow prone positioning and prevention of maternal movement during HIFU exposures.

On the day of exposure to HIFU or sham (“day 0”), general anaesthesia was induced with alfaxalone 3 mg/kg (I.V., Alfaxan, Jurox) and maintained with isoflurane (inhaled, 1.5–2.5% in 4:1 O_2_:N_2_O). Ewes were mechanically ventilated and maternal oxygen saturation and end-tidal carbon dioxide (EtCO_2_) were monitored non-invasively; Maternal SpO_2_ was maintained >94% and EtCO_2_ was maintained at <6%. The ewe was maintained in left lateral tilt while anaesthetised. Mechanical ventilation pauses of up to 90 s were required during HIFU exposures, because respiratory movement could lead to mistargeting. To ensure maternal and fetal wellbeing, mechanical ventilation would be resumed before the end of a HIFU exposure series if maternal EtCO_2_ rose above 8% or SpO_2_ fell below 94%. Matched ventilation pauses were performed in both sham and HIFU exposed animals. The median duration of anaesthesia was 147 min (range 142–161 min) in the control group and 144 min (range 128–165 min) in the HIFU groups (p = 0.45).

### Preparation of maternal abdominal skin and establishment of an acoustic window

Following induction of anaesthesia, maternal abdominal skin was first shaved with clippers to remove wool, then washed with iodine scrub solution (Vetasept: 7.5% povidone iodine, Animalcare Ltd.) to remove dirt and lanolin, and washed again with plain water. The remaining hair was chemically depilated (Veet® hair removal cream, Reckitt Benckiser), the skin was washed for a final time with plain water, and then wetted with degassed water. As HIFU needed to be applied to animal tissue through a good acoustic window, not air, a “water bag” was placed in contact with the maternal skin. This was a customised bag made from polythene, diameter 210 mm, filled with degassed water and suspended from an arm on the mechanical gantry arm (Figure s2). Trapped air between the plastic and the skin was removed, until no remaining air pockets were identified visually. If the water bag was repositioned, the layer of degassed water on the skin was replaced, and air pockets were again removed. The use of a water bag allowed the HIFU transducer to be positioned between 20–39 mm above the maternal skin.

In an attempt to reduce the subjective appearance of skin irritation observed in the first ewe (HIFU exposed), the interval between shaving (in unanaesthetised ewes) and chemical depilation (following induction of anaesthesia) was changed from 5–10 min to 24–48 h in the remaining 11 ewes. In animals 1–3 (HIFU exposed), the water in the water bag was taken cold from the tap but not temperature controlled; it was degassed before the start of HIFU exposures and only again if cavitation was seen in the water during HIFU exposures. In animals 4–6 the water was cooled with ice sealed in plastic and was degassed before each HIFU exposure series. The water was held at 19.7–26.3 °C and continuously degassed to give dissolved oxygen levels between 0.8–2.8 mg.l^−1^. No water bag was used in animals 7–12 as they were sham exposed.

### Assessment of materno-fetal wellbeing during HIFU/sham exposures

Materno-fetal Doppler studies, as described above, were repeated at three timepoints during the period of anaesthesia (Figure s1): (i) prior to the application of HIFU or sham exposures (0.5 h after induction of anaesthesia), (ii) at the midpoint of HIFU or sham exposures (1.5 h after induction), and (iii) at the end of HIFU or sham exposures (2.0 h after induction). Maternal heart rate, respiratory rate, oxygen saturation and end tidal CO_2_ were measured non-invasively with a veterinary anaesthetic monitor (VM-2500, Viamed).

### HIFU protocol

HIFU was applied through intact maternal skin, acoustically coupled using the degassed water-filled bag suspended from an arm on a positioning gantry. The Sonic Concepts H148MR transducer used (frequency 1.66 MHz, 64 mm diameter, 63 mm focal length, 19 mm central aperture for ultrasound imaging, focus diameter 1.2 mm, focus length 8.9 mm) was held in position within the water bag on an automated 3D positioning gantry (Figure s2). The transducer was driven by a signal generator via a power amplifier (A300, E&I Ltd, Rochester, NY). A laptop computer was used to run a graphical user interface (MATLAB R2013a, Mathworks) to control and log the automated gantry position, signal generator settings, and timing of exposures. Target vessels were identified using a 4–10 MHz phased sector array diagnostic ultrasound transducer (P10–4, Z.one Zonare ultrasound system, Mindray, China) centrally mounted behind the HIFU transducer. A 30 minute planning phase for placentome vessel mapping was allowed prior to the start of HIFU exposures, but after induction of anaesthesia and preparation of the maternal skin.

HIFU exposure conditions were: 4–7 exposures of 5 s duration spaced 5 s and 2 mm apart, electrical power 30–80 W. The electrical power used was selected based on the depth of the target beneath the maternal abdominal skin, with the intention of delivering estimated *in situ* intensity <5.0 kW.cm^−2^ to the target tissue (associated with excessive soft tissue damage). During the course of the experiment, reduction in electrical power when exposing shallower targets was also used as a way of reducing the rate of maternal skin burns. Electrical power levels selected for the target depths were as follows: ≥ 25.0 mm =78 W; 20.0–24.9 mm = 62 W; 15.0–19.9 mm = 50 W; 10.0–14.9 mm = 40 W; ≤ 9.9 mm = 32 W. Free-field measurements of peak focal pressure were made with a calibrated hydrophone up to 20 W (electrical) values up to 100 W were calculated by extrapolating those data. Estimated *in situ* intensity values were obtained by derating the free field intensity using the average abdominal wall attenuation and placentome attenuation coefficients which were measured using finite amplitude insertion measurements in an additional part of this study^16^. The depth of the target vessel was re-measured from saved color flow Doppler imaging of the vessel for calculation of estimated *in situ* intensities described in this study.

HIFU exposures were merged to form a single linear track, across the vessel. In order to reduce the risk of vessel haemorrhage, additional safety protocols were added to previously published protocols^3,17^. These were: (i) all exposure tracks were planned to start and finish in placental soft tissue bordering the echo lucent area from which vessels arise; (ii) the movement of the mechanical gantry arms was restricted to axes at 90° to the direction of sound/vertical midline of the ultrasound image. As previously, no HIFU exposure series were not used in vascular targets in which fetal parts were seen in the post focal region.

The target vessels were selected based on their ease of targeting with B mode and colour Doppler ultrasound, and position within the uterus (<4 cm from the skin). Six vessels were targeted in ewes 2–6; 4 vessels were targeted in ewe 1. Each was assessed before, and immediately after, HIFU exposure using colour Doppler in the same 3D position, controlled by the automated gantry, with 3 s video clips being recorded. Treatment success was defined as no detectable flow seen on colour Doppler after treatment using the lowest velocity scale setting (−6.3–6.3 cm.s^−1^) and the highest gain setting without causing colour saturation. After a failed occlusion, the vessel was re-exposed, unless as a result of the initial exposure series: (i) there was evidence of a maternal skin changes; (ii) there was suspicion of fetal or other maternal injury; (iii) there were mechanical or software errors which could not be immediately overcome. The maternal skin was inspected and photographed for evidence of skin changes after completion of each series of HIFU exposures. Skin damage which was present prior to HIFU exposures was not included. Superficial skin reddening which blanched with pressure and was resolved fully within 24 h was described as erythema. Skin damage which met any of the following criteria was described as a skin burn: (i) redness which persisted beyond 24 h, (ii) redness which did not blanche with pressure, (iii) white, tan or black skin discolouration, (iv) evidence of blistered skin, or (v) if exudate was associated with the skin damage.

### Postoperative care

Following completion of planned HIFU exposure series, animals were recovered from anaesthesia and monitored until day 21. Animals were housed in individual pens in an outdoor barn (light and dark cycle dependent on seasonal variation) with *ad libitum* access to hay, nuts, mineral lick and water. After five days, animals were transferred to communal pens in an outdoor barn with access *ad libitum* to hay, mineral lick and water; nuts were provided daily.

Animals were checked daily for signs of maternal pain or distress, infection or poor healing of skin burns, problems with mobility, bladder or bowel function, preterm labour, rupture of membranes or vaginal bleeding. Maternal analgesia (Carprofen 1.4 mg.kg^−1^ S.C., Rimadyl^TM^, Pfizer Ltd.) was administered if signs of maternal pain or distress were evident based on behaviour, facial expressions, stance or feeding patterns.

### Assessment of materno-fetal wellbeing following HIFU/sham exposures

Materno-fetal Doppler studies, as described above, was performed on a daily basis for the first five days after HIFU or sham exposures, and every fifth day thereafter. Fetal biometry, as described above, was repeated on the fifth day after exposures, and every five days thereafter (Figure s1). The formula used to calculate fetal growth rates was:

$${\bf{Fetal}}\,{\bf{growth}}\,{\bf{rate}}=(\mbox{''}{\bf{Day}}\,{\bf{20}}\mbox{''}\,{\bf{value}}\mbox{--}\mbox{''}{\bf{Day}} \mbox{-} {\bf{1}}\mbox{''}{\bf{value}})/(\mbox{''}{\bf{Day}}\,{\bf{20}}\mbox{''}\,{\bf{age}}\mbox{--}\mbox{''}{\bf{Day}} \mbox{-} {\bf{1}}\mbox{''}\,{\bf{age}})$$

where *value* represents the biometric measurement on the specified day for which growth rate was calculated (in mm), and *age* is the gestational age given in days.

### Post mortem examination

On “day 20”, the ewe and fetus were euthanised for post-mortem examination and tissue collection under schedule one of the UK Animals (Scientific Procedures) Act 1986. A slow IV injection into the maternal jugular vein of 120 mg.kg^−1^ pentobarbitone sodium (Pentoject®, Animalcare Ltd., York, UK) was used for this purpose. Air embolus into the jugular vein was used as a secondary confirmation of death. Maternal cardiac activity was auscultated with a stethoscope and death was confirmed on cessation of cardiac and respiratory activity. Cessation of fetal cardiac activity was confirmed with diagnostic ultrasound. A systematic inspection of tissues was performed at post mortem examination and a photographic record was compiled of damage to: (i) maternal skin, (ii) maternal rectus sheath, (iii) anterior and posterior surface of the uterus, (iv) fetal skin (dorsal, ventral, left and right lateral views), and (iv) maternal bladder and bowel. Blood samples were drawn into sterile syringes from the umbilical vein (0.5 ml each). The haemoglobin concentration of the blood was determined using an ABL80 Flex analyser (Radiometer, Denmark). All placentomes were excised, bisected and examined for evidence of tissue damage, weighed and classified by morphological type. In HIFU exposed animals, all placentomes with evidence of tissue damage were retained for histological examination. An additional 6 placentomes, randomly selected, were retained from each sham-exposed animal. Collected tissues were immersion fixed in 10% neutral buffered formalin for five days before embedding in paraffin wax. Eight micrometre sections were stained with haematoxylin and eosin (H&E) to provide a detailed view of damaged and undamaged tissues and Phosphotungstic Acid-Haematoxylin (PTAH) stains to identify areas of fibrin deposition. Measurements taken from histological sections were not corrected for shrinkage, as whether shrinkage rates of damaged and undamaged tissue was uncertain. Measurements therefore represent a minimum size, which may have been reduced artificially by fixation.

### Statistical analyses

All statistical analysis was performed in SPSS (version 22, IBM, NY, USA). Graphs were drawn in GraphPad Prism (version 6, GraphPad Software, Inc., San Diego CA, USA). Statistical significance was accepted when p < 0.05 for all tests, although where applicable individual p values are presented in graphs and tables.

Continuous data were assessed for normality using the Shapiro-Wilkes test. Descriptive analysis was performed using mean ± standard error of the mean (SEM) for normally distributed biometric data; mean ± standard deviation (SD) was used for normally distributed ultrasound data. Median ± 95% confidence intervals (95% CI) were used for non-parametric continuous data, unless otherwise stated. A two-tailed unpaired Student’s *t*-test was used to compare means (normally distributed) or a Mann-Whitney U-test was used to compare medians (non-parametric data).

To assess the effect of HIFU treatment on a continuous dependent variable when additional independent variables needed to be considered, two-way ANOVA testing was used. A repeated measures two-way ANOVA was used to investigate the change in the dependent categorical variable due to the interaction of time with HIFU treatment; a standard two-way ANOVA was used if the second independent categorical variable was not time. If a significant effect or interaction was identified post hoc testing as applicable was performed to identify the source of variation.

Proportions of categorical data are described as numerator over denominator, with a percentage value given. A chi-squared test was used for univariate analysis of categorical data, with additional calculation of odds ratios and relative risks and their associated 95% confidence intervals.

The datasets generated and analysed during the current study are available from the corresponding author on reasonable request.

## Results

### Efficacy of vascular occlusion

**Figure 1:**
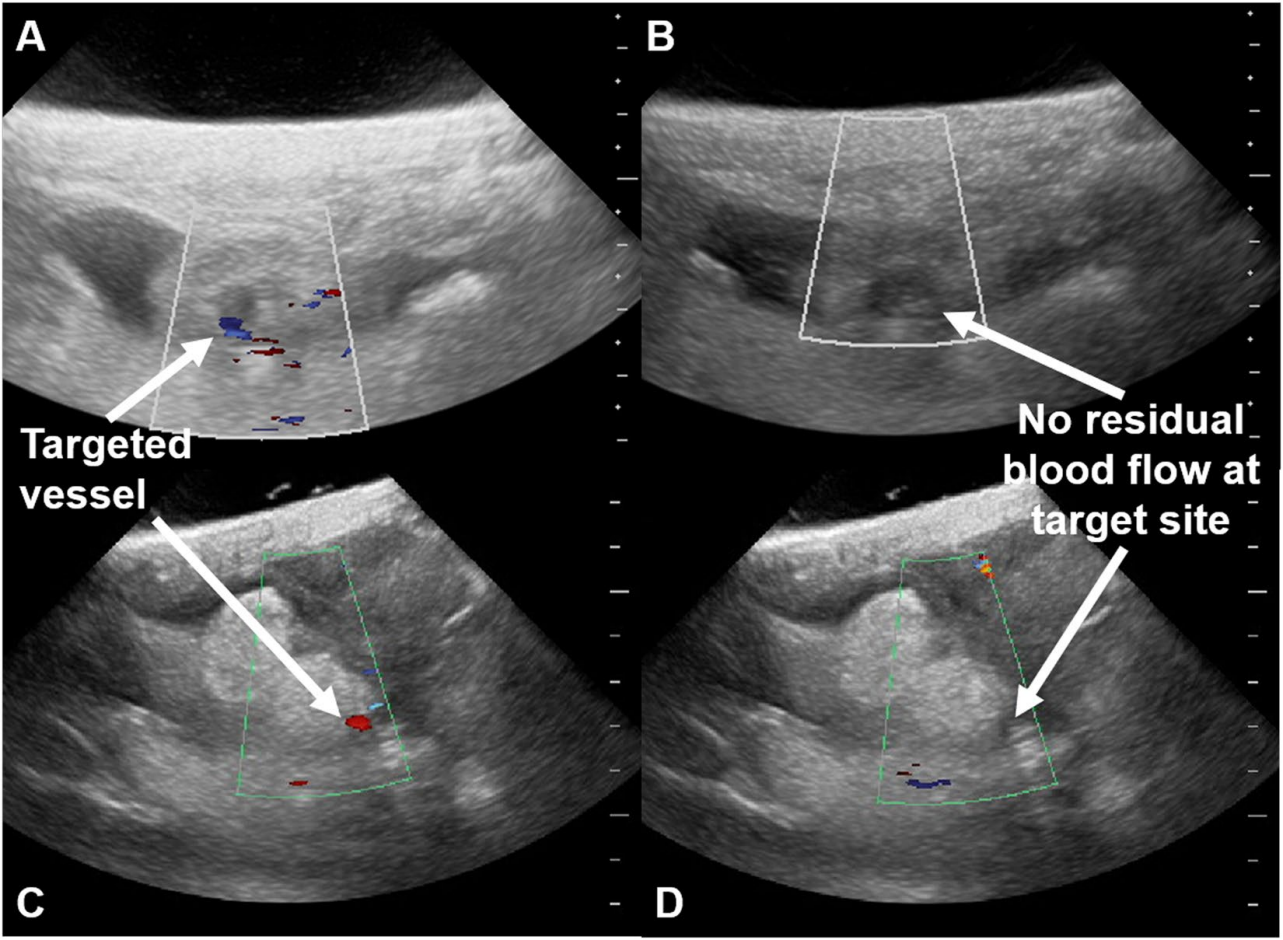
Colour flow Doppler imaging demonstrating successful placental vascular occlusion. (**A**,**C**) Pre-exposure colour flow Doppler imaging of a placentome. The intended vascular target is labelled. The colour flow Doppler gain is −25.0–25.0 cm.s^−1^ in image (**A**) and -13.0–13.0 cm.s^−1^ in image (**C**). Images were obtained using a 4–10 MHz phased sector array transducer (P10–4, Z.one Zonare ultrasound system, Mindray, China). (**B**) Post-HIFU exposure colour flow Doppler imaging of the placentome shown in image (**A**) demonstrating no colour flow Doppler signal “no flow” within the targeted vessel. The HIFU exposure series which resulted in occlusion comprised 6 transdermal exposures, 2 mm spaced, 5 s duration, 5 s interval, estimated *in situ* intensity 3.0 kW.cm^−2^. (**D**) Post- HIFU exposure colour flow Doppler imaging of the placentome shown in image (**C**) demonstrating “no flow” within the targeted vessel. The HIFU exposure series which resulted in occlusion comprised 6 transdermal exposures, 2 mm spaced, 5 s duration, 5 s interval, estimated *in situ* intensity 4.1 kW.cm^−2^.

Based on comparison of pre- and post-HIFU exposure colour flow Doppler imaging, 31 of 34 vessels were successfully occluded (91%), examples of which are shown in Fig. 1. Regarding the successful occlusions, 29/31 vessels were occluded in a single exposure series; 2/31 vessels were occluded following second HIFU exposure series (a “retreatment”), giving a total of 36 exposure series of the 34 vessels. In 5 of 36 exposure series, occlusion was not achieved. Further details of the vascular targets, exposure characteristic and outcomes for each HIFU exposure series are given in Table s1.

In 2 of the 5 HIFU exposure series which resulted in failed occlusion, problems with control of movement of the mechanised gantry arm resulted in HIFU not being delivered as planned (i.e. multiple exposures into a single 3D location), a situation which had previously resulted in vessel haemorrhage. In both cases this was recognised and the HIFU exposure series was manually stopped before several HIFU exposures could be delivered to one location, thus preventing vessel haemorrhage. However, in both cases, retreatment was contraindicated by maternal skin damage. In a further 2 HIFU exposure series in which occlusion failed, there was a mistargeting of 10–14 mm in depth. In 1 of these a maternal skin burn prevented retreatment; in the other correction of the targeting error resulted in successful occlusion. In the final exposure series which resulted in a failed occlusion there was no obvious cause of failure (estimated *in situ* intensity 3.9 kw.cm^−2^). Retreatment with the similar exposure conditions (estimated *in situ* intensity 4.1 kw.cm^−2^) resulted in successful occlusion.

**Figure 2:**
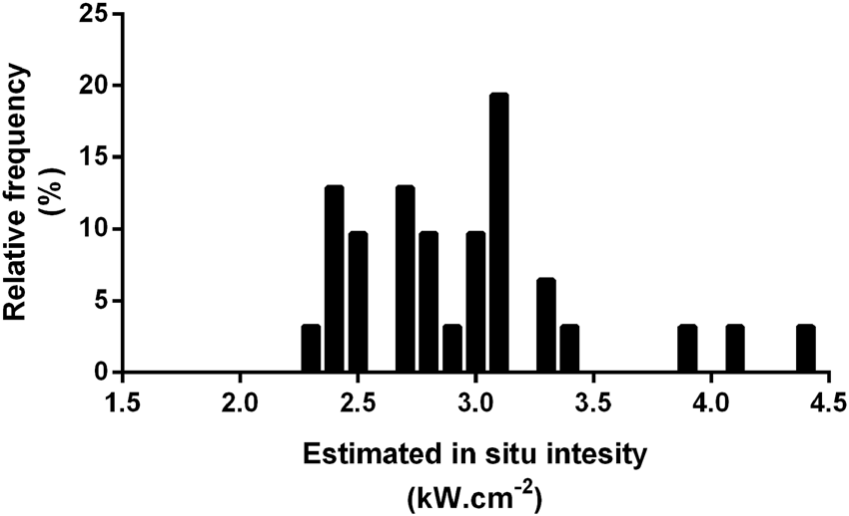
Estimated *in situ* intensity resulting in successful occlusion. The histogram shows the relative frequency of estimated *in situ* intensities which produced successful vascular occlusion.

The mean depth of the target vessels was 26 mm (SD ± 9 mm). The range of *estimated in situ* intensities delivered to the target vessels to produce successful occlusion was 1.3–4.4 kW.cm^−2^. The distribution of estimated *in situ* intensities which produced successful occlusions is shown in Fig. 2. As detailed above, only one occlusion failed when the planned HIFU energy was delivered in the intended manner, so a comparison to *in situ* intensities of failed occlusions is not possible. If a vessel was occluded in the first exposure series, and there was no equipment error, the median time to complete a HIFU exposure series and determine whether occlusion had succeeded or failed was 101 s (range 74–232 s). The two vessels where retreatments were required took longer (including the time for the first exposure series, identification of failed occlusion and contributing factors): 305 and 1087 s. Failed occlusions following equipment error were identified quickly: 49 and 124 s (Table s1). No instances of vessel haemorrhage were seen using colour Doppler imaging.

### Persistence of vascular occlusion

Twenty days following HIFU occlusion, 34 placentomes with evidence of tissue damage were retrieved at post-mortem examination in animals exposed to HIFU. While one to one correlation of exposed placentomes to colour Doppler findings and HIFU exposure conditions was not possible due to the time elapsed since exposure, both the total number, and the number per animal, matched the number of exposed vascular targets. No damage was seen in the sham exposed placentomes. Histological examination confirmed that there was no evidence of vessel haemorrhage.

**Figure 3:**
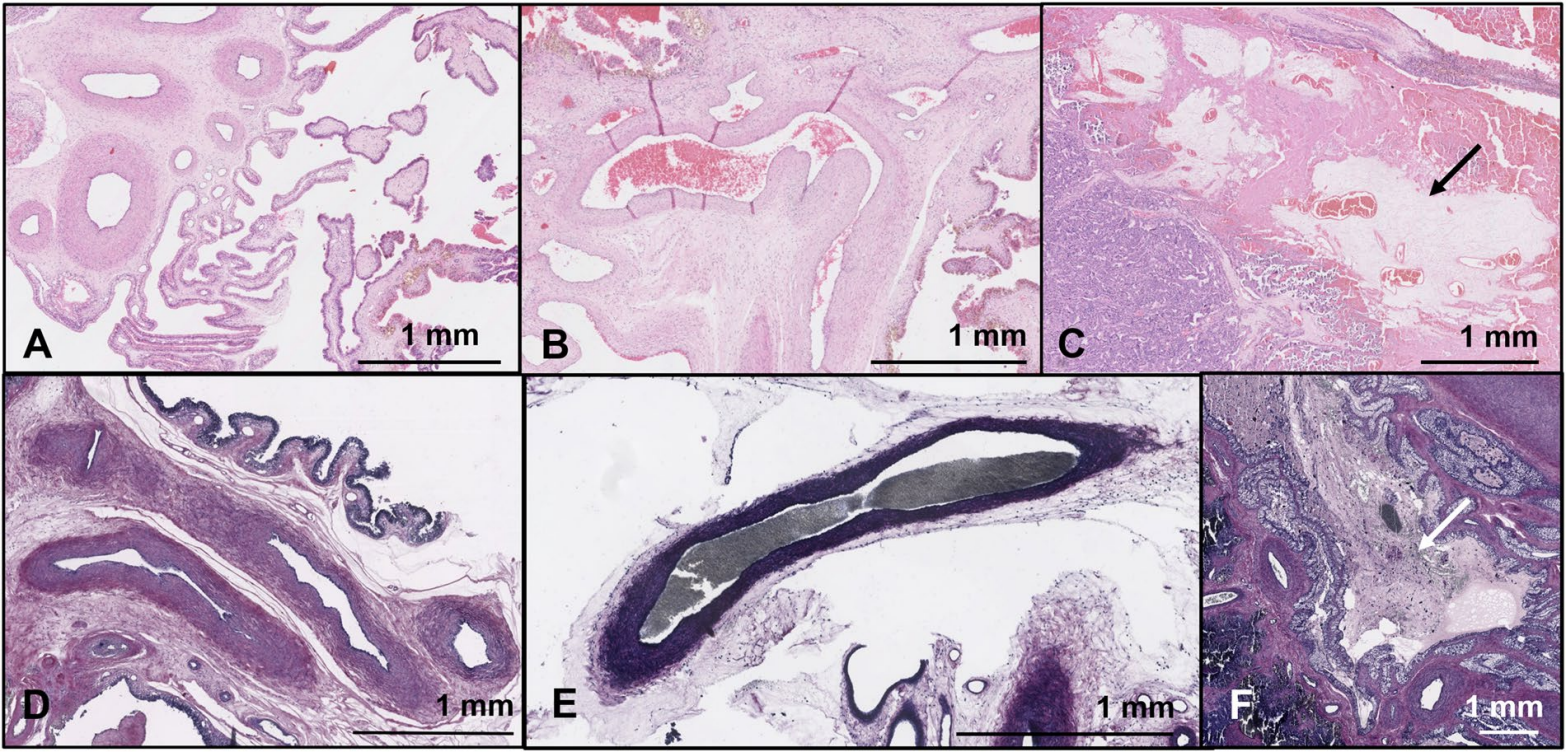
Histological confirmation of vascular occlusion after 21 days. (**A**) H&E stain of fetal vessels in a sham exposed placentome. The lumen of the vessels are open and unobstructed, suggesting they remain patent, and the tissue around the vessels shows regular pink staining of normal connective tissue. (**B**) H&E stain of fetal vessels in an exposed placentome where colour Doppler imaging suggested occlusion. While the vessel lumen is not collapsed, there are trapped erythrocytes within the lumen suggestive of occlusive clot. (**C**) H&E stain of fetal vessels in an exposed placentome where colour Doppler imaging suggested occlusion; again the lumen are occluded with trapped erythrocytes. The black arrow marks pale, irregular staining in the tissue around the vessels denoting vacuolar degeneration. (**D**) PTAH stain of fetal vessels in a control placentome. The lumen are open and unobstructed and the connective tissue around the vessels has regular purple staining of normal collagen. (**E**) PTAH stain of a fetal vessel exposed to HIFU and suggested to be occluded by colour flow Doppler studies. The lumen is stained black, indicating that it is occluded by organised fibrin, a definitive appearance of occlusive clot. (**F**) PTAH stain of HIFU exposed fetal vessels: there is again black staining in the lumen, but also regions of irregular, pale staining in the collagenous regions surrounding the vessels (white arrow), matching the H&E finding of vacuolar degeneration.

Trapped erythrocytes within vessel lumina, suggestive of occlusive clot, were found in 30/34 placentomes which had been exposed to HIFU (Fig. 3); no trapped erythrocytes were found in the lumina of the three vessels in which colour Doppler indicated failed occlusion. In one placentome, the tissue could not be sectioned adequately to visualise the vessels (occlusion predicted by colour Doppler). PTAH staining confirmed the presence of organised fibrin in those vessels in which trapped erythrocytes were seen (Fig. 3). Again, while it was not possible to directly match the colour Doppler and histological findings, the number of placentomes with occluded vessels identified in each animal by colour Doppler and histology matched, apart from one animal, in which one placentome could not be examined histologically. The mean diameter of occluded vessels was 1.4 mm (range 0.3–3.3 mm, without correction for tissue shrinkage during histological processing). There was no evidence of trapped erythrocytes within vessel lumina of the 36 placentomes from sham exposed animals examined.

Vacuolar change, suggestive of degeneration of the connective tissue surrounding vessels (Fig. 3) was found surrounding occluded vessels in 24/30 placentomes where there was histological evidence of occlusion. There was no evidence of similar vacuolar degeneration surrounding the 3 vessels for which occlusion failed, nor surrounding vessel lumina taken from sham-exposed placentomes.

### Fetal outcomes

All fetuses survived (undelivered) to the end of the follow-up period. There were no instances of preterm delivery or preterm rupture of membranes. At post mortem examination of the fetuses, there was no evidence of burns or injury, meconium staining of liquor, nor haemorrhage from either placental vessels or fetal structures. The fetal haemoglobin concentration of cord blood was 12.4 g.dl^−1^ in sham exposed and 13.2 g.dl^−1^ HIFU exposed fetuses at the time of post mortem examination (p = 0.49).

The fetal growth rate, based on changes in EFW, BPD, AC or FL, estimated by ultrasound, was not different between exposure groups. The fetal weight, biometry and measures of asymmetric growth were not different between sham and HIFU exposed animals after completion of 20 d follow-up when measured at post-mortem examination (Table 1).

**Table 1:** Fetal biometry, growth rate and indices of asymmetric growth.

	Sham Exposed			HIFU Exposed			*p value*
Maternal weight (kg)	46.0	±	2.7	46.0	±	1.5	1.0
Gestational age (d)	136.5	±	2.5	135.3	±	2.4	0.77
Male: Female ratio	1:1			1:2			0.26
***Fetal Biometry***							
Fetal weight (kg)	3.8	±	0.3	3.3	±	0.2	0.28
Fetal BMI (kg.m-2)	15.6	±	0.6	16.6	±	0.8	0.40
Bipareital diameter (cm)	6.6	±	0.3	6.4	±	0.2	0.66
Abdominal circumference (cm)	30.5	±	0.8	31.3	±	1.1	0.58
Femur length (cm)	9.2	±	0.5	8.9	±	0.5	0.73
Bipareital diameter: abdominal circumference ratio	0.22	±	0.008	0.21	±	0.006	0.36
Brain: liver ratio	0.55	±	0.02	0.58	±	0.04	0.54
Adrenal: body weight ratio	0.18	±	0.04	0.17	±	0.03	0.93
***Fetal Growth Rate***							
Bipareital diameter (mm.d^−1^)	0.9	±	0.3	0.8	±	0.3	0.90
Abdominal circumference (mm.d^−1^)	3.0	±	0.9	3.6	±	0.8	0.43
Femur length (mm.d^−1^)	1.6	±	0.6	1.8	±	0.6	0.77
Estimated fetal weight (g d^−1^)	107	±	27	90	±	20	0.34

Values represent mean ± SEM of maternal characteristics, fetal biometry, indices of asymmetric growth measured at post-mortem, 20 days after HIFU (n = 6) or sham (n = 6) exposures of placental vasculature and general anaesthesia. The mean ± SD of the calculated fetal growth rate of biparietal diameter, abdominal circumference, femur length (measured by ultrasound) and estimated fetal weight (calculated) for fetuses in the HIFU (n = 6) and sham (n = 6) groups during the 21 day follow-up period are also shown. Differences between groups were assessed with a two-tailed student’s t-test and no significant differences were found (*p* values shown in table).

**Figure 4:**
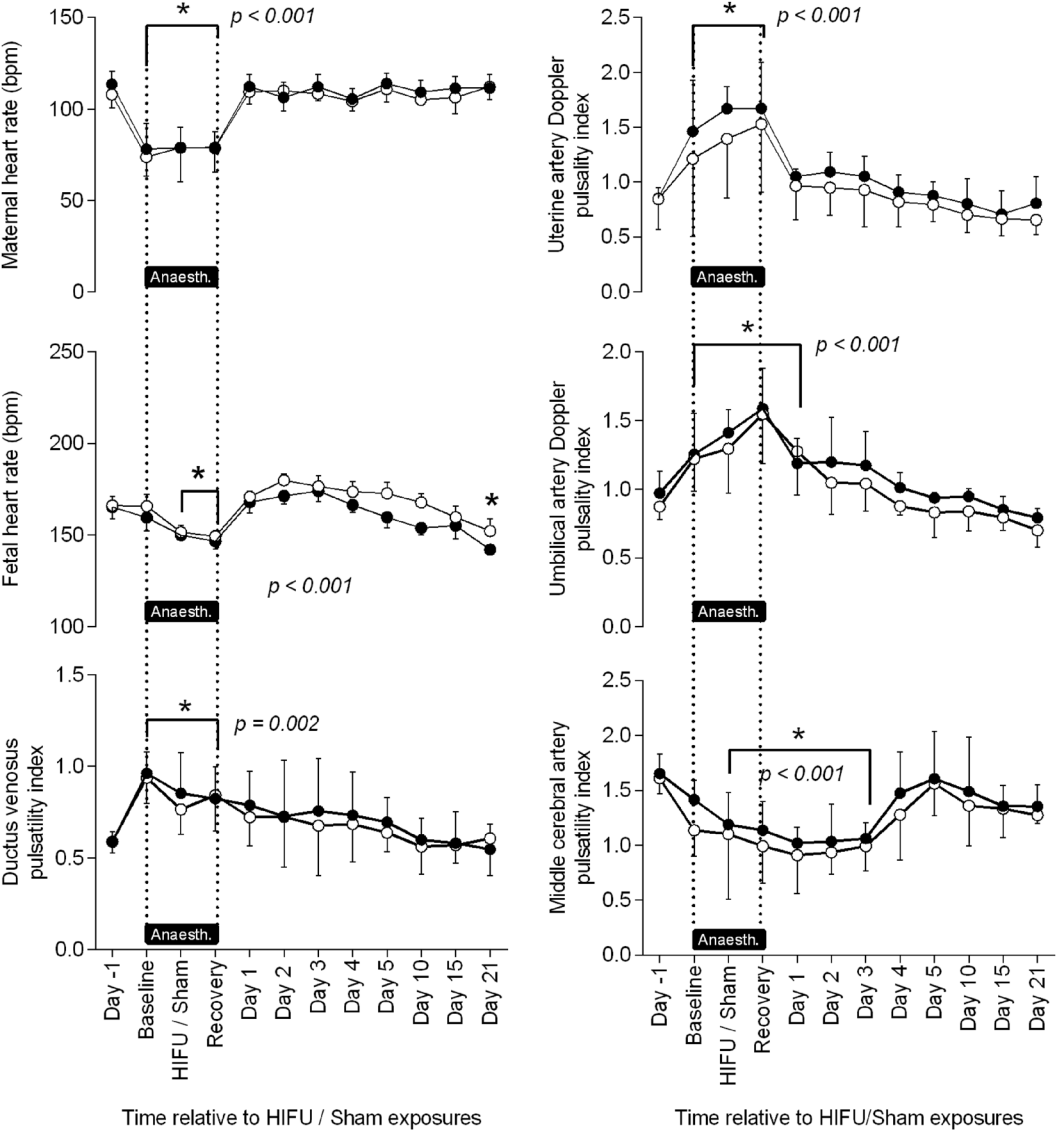
Fetal Doppler indices before, during and after HIFU/sham exposures. Values represent the mean ±SD of maternal and fetal heart rate, maternal uterine and fetal umbilical and middle cerebral artery and Ductus venosus pulsatility index, before, during and after transdermal HIFU (closed circles; n = 6) or sham (open circles; n = 6) HIFU placental vascular occlusion under general anaesthesia. Statistical significance was assessed using a repeated measures two-way ANOVA with post hoc Tukey’s test; *denotes a significant difference of time with respect to day −1, with exact p values given on the graph. There was no significant difference of the effect of treatment.

During experimental procedures (“day 0”), there was a statistically, but not clinically, significant decrease in fetal heart rate, which occurred during the first 60 minutes of general anaesthesia, in both exposure groups. Pre-procedure (“day −1”), there was no difference in resistance to umbilical flow between groups: the mean ± SD UA-PI in the sham group was 0.87 ± 0.16 and in the HIFU group was 0.97 ± 0.16 (ns, p = 0.21). Following induction of anaesthesia, there was a progressive rise in the UA-PI in both groups with duration of anaesthesia, which remained statistically significant for 24 h. This returned to baseline after 3–4 days (Fig. 4). The MCA-PI decreased during anaesthesia and HIFU or sham exposures, and this persisted for the first 3 days of the follow-up period (Fig. 4). The DV-PIV was elevated during the period of anaesthesia but was not significantly increased following the reversal of anaesthesia (Fig. 4).

### Placental size and morphology

There were no differences in placentome size or morphology between HIFU and sham exposed animals. The total placentome weight in sham-exposed animals was 297 ± 23 g and 286 ± 22 g in HIFU-exposed animals (p = 0.76). The ratio of number of A/B to C/D type placentomes was 27 ± 15 in sham exposed and 19 ± 12 in HIFU exposed animals (p = 0.71).

### Maternal outcomes

All ewes were alive at the end of the planned HIFU exposure series and survived to the end of the follow-up period. Based on daily systematic observation of maternal movements and gait, food intake, passage of urine and faeces, there was no suggestion of injury to the spinal cord, bladder or bowel.

**Figure 5:**
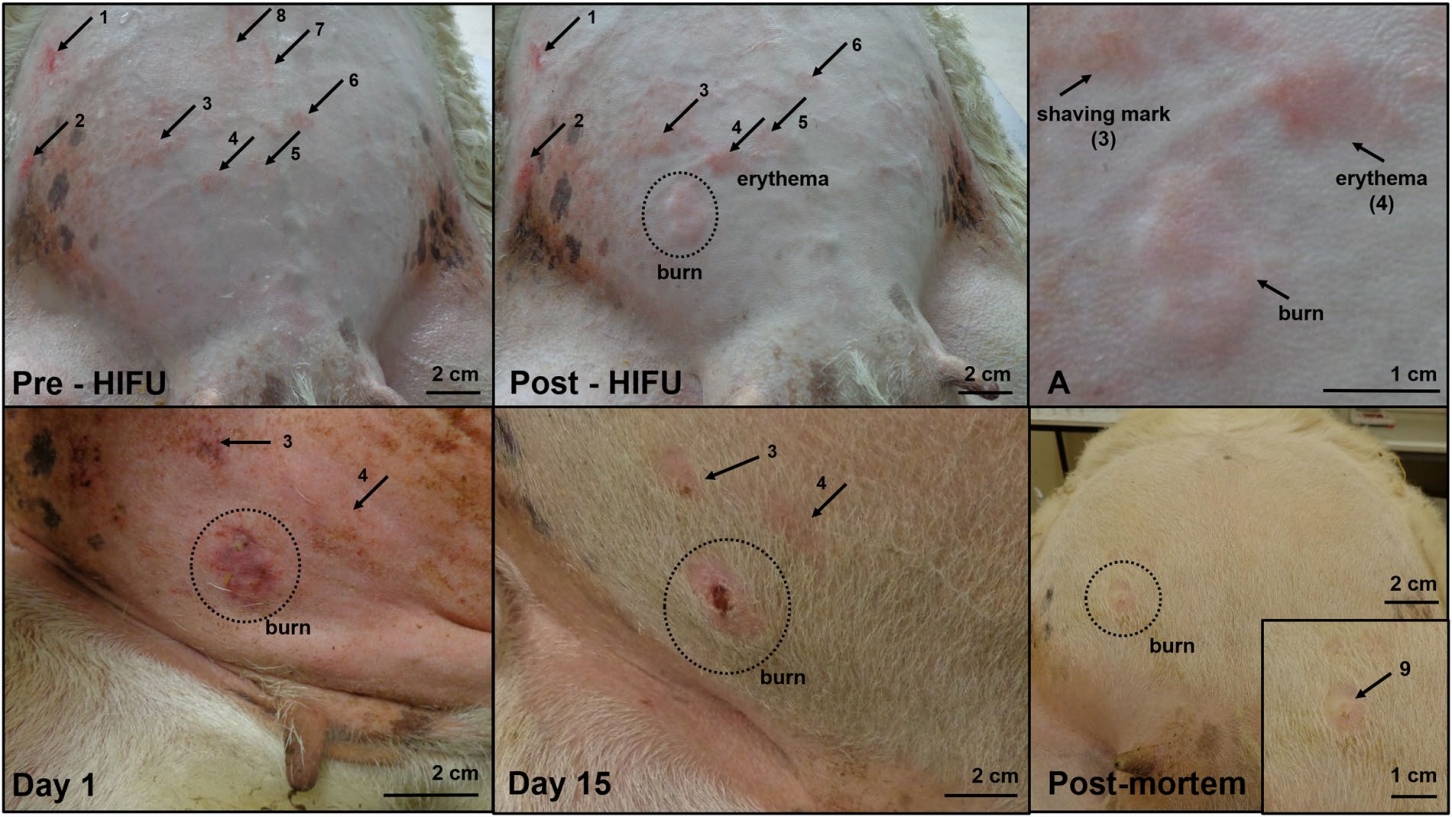
Maternal skin burn healing. Pre-HIFU: this panel shows the maternal abdomen after shaving and depilation (24 h apart) before exposure to HIFU. Arrows 1–8 mark areas of skin irritation or damage as a result of shaving or chemical depilation. Post HIFU: the maternal abdominal skin is shown after completion of 6 HIFU exposure series. The circled area is a skin burn which was not present before HIFU; the arrow marked 4 shows an area of skin irritation which has enlarged, and appears to be erythema. Areas of skin irritation 1–3 and 5–6 remain unchanged, 7–8 are no longer visible. An enlargement of the new areas of skin damage is shown in panel A. Day 1: the burn has darkened and has a small yellow area of fibrinous exudate. There is now no clear evidence of skin damage at location 4, supporting the classification of erythema; there appears to be an area of persistent skin irritation at location 3, which is starting to develop a fibrinous scab. Day 15: The burned area has formed a fibrinous scab with no sign of infection. A small area of fibrinous scab remains at the area of skin irritation (3); and while there is no skin damage at site 4, the regrowth of hair is altered. Post-mortem: by day 21, the area of skin irritation has healed, and the burn is a raised while area of tissue (shown enlarged in the inset, marked by arrow 9).

There were 4 maternal skin burns as a result of a total of 36 HIFU exposure series, affecting 3 of 6 HIFU exposed ewes. There were 10 instances of maternal skin erythema, which were fully resolved within 24 h, affecting 5 ewes. The last ewe in the HIFU exposed group had no skin burns or erythema. The 4 maternal skin burns did not show signs of infection during the follow-up period and had begun to heal. Examples of skin burns and erythema are shown in Fig. 5.

The majority (3 of 4) skin burns occurred when the highest HIFU energy output was used, although a reduction in target *in situ* intensities alone produced no obvious improvement in skin damage rates. Skin burns occurred when target *in situ* intensities of 2.7–4.4 kW.cm^−2^ were used; skin erythema occurred when target *in situ* intensities of 1.3–3.4 kW.cm^−2^ were used. Skin burns also occurred at shallower target depths: 3 of 4 burns were in exposure series with a target depth ≤15 mm. However, 10 instances of skin damage occurred when the target depth was >15 mm. Skin damage occurred in both the heaviest (50 kg) and lightest (33 kg) ewes.

Maternal analgesia was given in the ewes (n = 3) with skin burns for 5 days post HIFU as per veterinary instruction, despite not displaying signs of pain during these days or at any later point. In the other 3 animals in which there were no burns, animal pain scores also were normal. At post mortem examination, there was no visual evidence of damage to the rectus sheath or uterus, faecal peritonitis, lesions on the bowel, uroperitoneum lesions on the bladder, or injury extending into the retroperitoneum, although the spine was not formally examined.

During the experimental procedures, there was a decrease in maternal heart rate, which occurred during the first 30 minutes of general anaesthesia and persisted until its reversal. Pre-procedure (“day −1”), UtA-PI for both groups was <1.0. Under anaesthesia, there was a progressive rise in the UtA-PI with time. The UtA-PI was again <1.0 by 24 h post procedure and remained stable thereafter. These increases in UA-PI were not different between HIFU and sham treated groups (Fig. 4).

## Discussion

This study demonstrates a high rate of successful placental vascular occlusion in sheep, using HIFU applied through intact maternal skin. The efficacy of vascular occlusion (91%) approximates to that of our previous, transuterine, study^3^: 93%. The success rates of ultrasound guided HIFU vascular occlusion quoted in published literature varies from 63–100%, although there are only 5 relevant papers^18–22^. In our study, identification, targeting and confirmation of occlusion was successfully performed with colour flow Doppler ultrasound, integrated with the HIFU transducer, in vessels of clinically relevant diameters for human placental vasculature: arterio-venous anastomoses, the causative vessels of TTTS, are quoted in published literature between 0.9–2.1 mm^23^. There were no instances of vessel haemorrhage, nor fetal or uterine injury. We were also able to demonstrate persistent vascular occlusion 20 days after exposure to HIFU, using a fibrin-specific PTAH stain, which correlated well with findings of “no flow” on colour flow Doppler. This indicates the utility of colour Doppler as a non-invasive, real-time method of confirming HIFU mediated vascular occlusion in this setting.

While HIFU ablation of placental tissue through intact maternal skin has previously been demonstrated^2,4^, this study is the first demonstration that selective placental vascular occlusion during sheep pregnancy could be achieved through intact skin. This is an essential prerequisite for its successful translation to human studies.

In this study, vascular occlusion was also achieved at lower *in situ* intensities than in the previous transuterine study^3,24^. In the animals in the present study, the median *in situ* intensity delivered to target vessels was 2.9 kW.cm^−2^ (IQR 2.5–3.1 kW.cm^−2^, compared to 5.4 kW.cm^−2^ (IQR 5.1–5.5 kW.cm^−2^) in the transuterine exposures. The initial decision to use high intensities was based on prior studies in which vascular occlusion had typically required high intensities, with the attendant risk of complications^6,7,25,26^, but in this study the intention was to avoid *in situ* intensities over 5.0 kW.cm^−2^ due to the excesses placentome damage seen and the associated risks of haemorrhage. In our previous transuterine study, unintentional delivery of excessive HIFU energy into a single tissue location due to equipment failure resulted in vessel haemorrhage^3^. In this instance, the linear track of exposures, starting and finishing in placental soft tissue was not completed, and the vessel wall was repeatedly exposed to high estimated *in situ* intensity exposures (6 exposures, 5 s duration, 5 s interval, estimated *in situ* intensity 5.4 kW.cm^−2^). In the present study, a combination of introducing (i) power to depth ratios, resulting in lower *in situ* intensities delivered to vessels, (ii) safety protocols requiring the linear track of HIFU exposures to start and finish in placental soft tissue, and (iii) manual stopping of planned HIFU exposure series in the event of non-movement of the mechanical gantry arm successfully prevented vessels haemorrhage. The technique of placing a linear track of exposures across a vessel has previously been described by members of our group, although in these studies ultrasound guidance was not used and testing was in a rodent animal model^6,7^. The ability to reduce the HIFU energy delivered to tissue and still achieve occlusion would also be expected to reduce the rate of complications related to high intensities, such as skin burns or thermal spread to adjacent structures^8^. As such, while the HIFU protocol used appears effective and safe, there is still scope for further refinement of the planning of exposure conditions before translation into human therapies.

There were no significant maternal or fetal injuries or obstetric complications identified, however maternal skin burns affected 3 of 6 ewes exposed to HIFU. A recent retrospective review of 9,988 patients treated with HIFU for conditions such as uterine fibroids, adenomyosis, placenta accreta, abdominal wall endometriosis and Caesarean scar pregnancy reported a 10.6% rate of adverse events. The rate of abdominal skin burns was 0.3% (26/9988) and 4/26 required surgical removal of necrotic tissue arising from the skin burns^27^. These rates of complications reflect the use of a commercially available HIFU therapy system and an established treatment protocol: they also rely on patient reporting of adverse events. They are lower than rates reported in smaller scale prospective studies, focused on developing treatment protocols, with planned follow-up of patient outcomes. Reported skin burn rates are between 4%^28–30^ and 10%^31^ and range from skin reddening^31^ to full thickness third degree burns^27,32^. This indicates the rate of maternal skin damage in our study was higher than expected from human studies.

The changes to the protocol in our study – reduction in skin irritation, cooling and degassing of the water in the water bag – did produce a reduction in, but not complete avoidance of, skin burns. In animal 1, none of these measures were used, and 3 of 4 (75%) exposure series resulted in skin damage. In animals 4–6, where all these measures were used, there were 4 of 18 (22%) exposure series caused skin damage. This is a relative risk of 0.24 (95% CI 0.08–0.69). Therefore, the actual rate of maternal skin burns was difficult to determine exactly since progressive changes to the HIFU treatment protocol were being made to reduce the number of skin burns. For this reason, the overall rate of skin burns does not reflect the likelihood of skin burns occurring if the final protocol had been used in all animals.

Importantly, we demonstrated the survival of mothers and fetuses for three weeks beyond the end of non-invasive HIFU exposures under general anaesthesia. Previous studies in animal models have not been designed to assess survival following HIFU exposure of the placenta^3–5,10^, although there are 2 case reports in humans of fetal survival and delivery following HIFU energy delivery into the intrauterine space (although not used on placental vessels)^1,33^. This represents a significant advance in demonstrating the potential of HIFU mediated placental vascular occlusion as a therapy suitable for use in human pregnancy.

Additionally, there was no evidence of fetal compromise resulting from HIFU exposures. There was consistent fetal growth velocity in both groups, and in utero fetal size was comparable with other invasive and non-invasive measures of fetal growth reported at corresponding points of gestation^14,34^. At post mortem, there was no difference in fetal weight, biometry, fat deposition or evidence of asymmetric. While even apparently significant hypoxic insults in late gestation do not always produce growth restriction, they are associated with asymmetric growth^35–37^. Notably, in HIFU exposed fetuses there was no difference in adrenal to body weight ratio, which has been shown following a 3 day reduction in sheep umbilical artery blood flow in late gestation^35^.

Pre-procedure, the mean fetal-maternal Doppler indices were within expected ranges from published sheep literature and human pregnancy at a corresponding gestational age^38–43^. The timing of the increase in resistance in the umbilical circulation matches the timing of the increase in resistance to flow in the maternal uterine artery, a known effect of isoflurane anaesthesia^44–50^. While there was no direct observation of arterial oxygenation in these fetuses, consideration of the values for arterial oxygen content and tissue delivery in our previous study^3^ would indicate that this was a fetal non-hypoxic period. Therefore, the changes in our fetuses simulate the classic findings of utero-placental dysfunction and fetal redistribution of blood flow preferentially to the central organs, despite hypoxia not being the underlying cause. This pattern of central redistribution of blood in response to EXIT surgery, in which the fetus was not hypoxic, has been described in humans^51^. Collectively, these findings suggest that changes fetal physiology were the result of general anaesthesia, rather than of HIFU exposures themselves, and all were fully reversible. This, in conjunction with the finding of maintained fetal growth, suggests no clinically significant effect on the utero-placental circulation as a result of the HIFU occlusion of placental vasculature.

In summary, transabdominal HIFU has been used to occlude sheep placental blood vessels of similar calibre to those seen in human placental affected by TTTS. There remains a risk of maternal skin burns which requires further refinement of the technique. The sheep fetus can withstand the challenges of anaesthesia – not a planned part of human treatment - and HIFU, and recover from these events without impact on fetal cardiovascular function or growth. It should be noted that the sheep fetuses were healthy and a human fetus may differ, or one already compromised fetus affected by TTTS may have reduced reserve to withstand HIFU vascular occlusion. Hence the principal aim of developing a non-invasive method to divide fetal circulations is to reduce the risks associated with the currently available invasive therapies and allow the option of earlier intervention without endangering the pregnancy before significant fetal compromise occurs.

## Electronic supplementary material

Supplementary figures and tables
