# Supplementary material for: Trans-abdominal in vivo placental vessel occlusion using High Intensity Focused Ultrasound
Source: Sci Rep. 2018 Sep 11;8:13631. doi: 10.1038/s41598-018-31914-4 (PMC6134117; doi:10.1038/s41598-018-31914-4)

**Title: Trans-abdominal *in vivo* placental vessel occlusion using High Intensity Focused Ultrasound**

**Authors:** Caroline J. Shaw^1,2^, Ian Rivens^3^, John Civale^3^, Kimberley J. Botting^1,4^, Gail ter Haar^3^, Dino A. Giussani^1,4^, Christoph C. Lees^2,5*^

**Supplemental Materials**


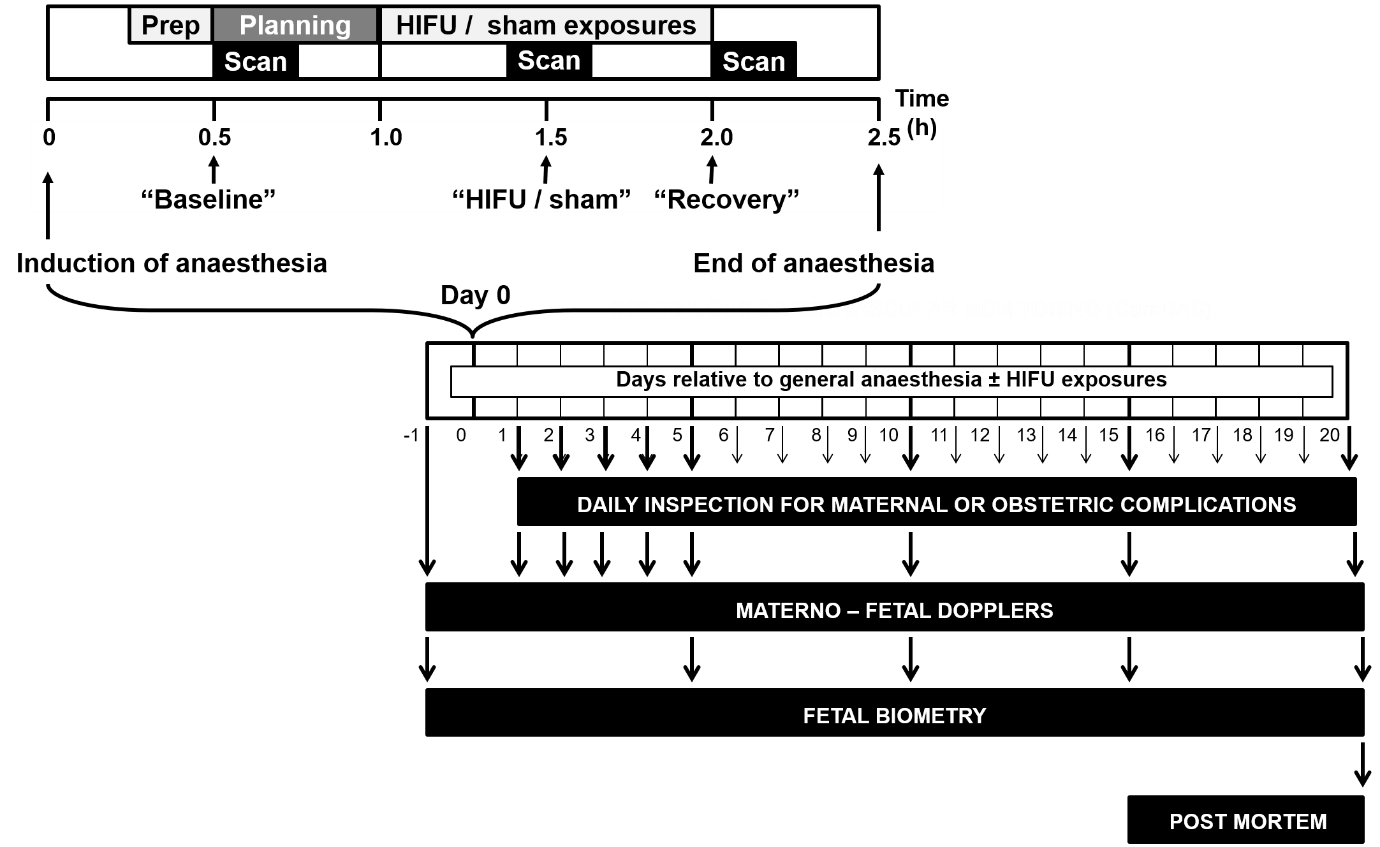


Figure s1: Timeline of experimental procedures and assessment of materno-fetal wellbeing

The schema shows the frequency and timing of daily inspections, materno-fetal ultrasound and post mortem examination, relative to “day 0”, when ewes underwent HIFU or sham exposures. The experimental timeline, divided into periods of experimental preparation, (“prep”), planning of vascular exposures, exposure to HIFU, materno-fetal ultrasound examinations and typical length of anaesthesia is also shown.


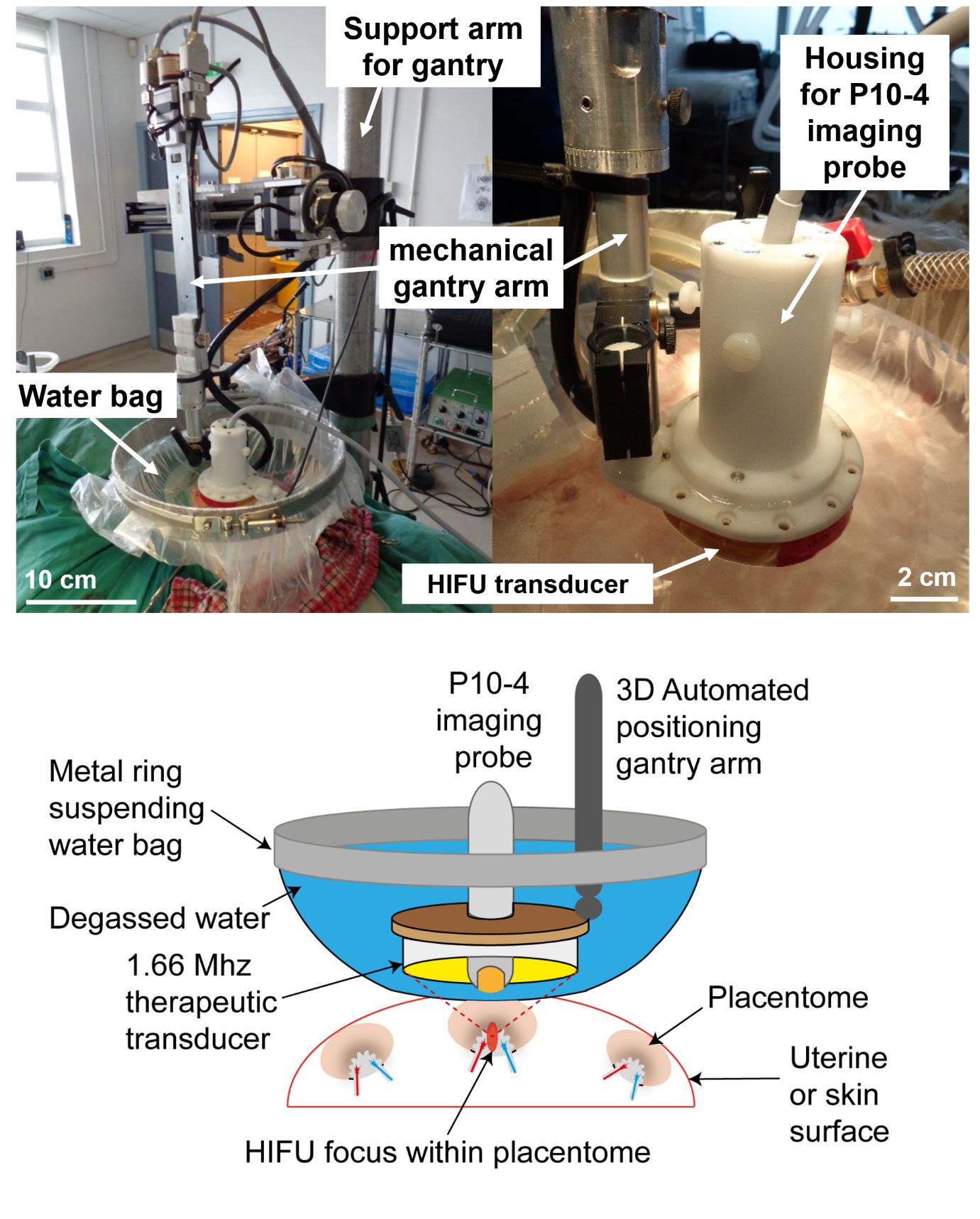


Figure s2: HIFU therapy system setup.

Top left: the picture shows the mechanical gantry arm and associated support structures in relationship to the water bag, HIFU and diagnostic ultrasound transducers. The ewe is lying supine, covered by surgical drapes (green), with maternal abdominal skin exposed only beneath the water bag. Top right: the picture shows an enlargement of the HIFU therapy transducer and the housing of the diagnostic transducer which allow integration of the two transducers, and their attachment to the mechanical gantry arm. Bottom: the diagram shows the arrangement of diagnostic and HIFU transducers in relationship to the gantry arm, the water bag, degassed water, and the maternal uterine or skin surface (not to scale).


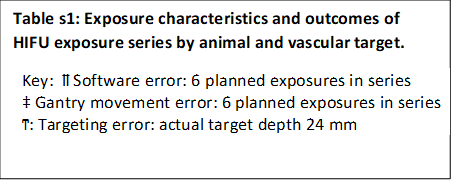

Supplement: Supplementary file 1 — Supplementary figures and tables [file 41598_2018_31914_MOESM1_ESM.docx]
